# Analytical Modeling of a Doubly Clamped Flexible Piezoelectric Energy Harvester with Axial Excitation and Its Experimental Characterization

**DOI:** 10.3390/s21113861

**Published:** 2021-06-03

**Authors:** Jie Mei, Qiong Fan, Lijie Li, Dingfang Chen, Lin Xu, Qingyang Dai, Qi Liu

**Affiliations:** 1 Institute of Intelligent Manufacturing and Control, Wuhan University of Technology, Wuhan 430063, China; meijiewl@whut.edu.cn (J.M.); fanqiong@whut.edu.cn (Q.F.); dfchen@whut.edu.cn (D.C.); daiqingyang@whut.edu.cn (Q.D.); liuqiqi@whut.edu.cn (Q.L.); 2 College of Engineering, Swansea University, Swansea SA1 8EN, UK; 3 State Key Laboratory of Advanced Technology for Materials Synthesis and Processing, School of Materials Science and Engineering, Wuhan University of Technology, Wuhan 430070, China; linxu@whut.edu.cn

**Keywords:** doubly clamped, flexible piezoelectric energy harvester, axial excitation, Euler–Bernoulli beam theory

## Abstract

With the rapid development of wearable electronics, novel power solutions are required to adapt to flexible surfaces for widespread applications, thus flexible energy harvesters have been extensively studied for their flexibility and stretchability. However, poor power output and insufficient sensitivity to environmental changes limit its widespread application in engineering practice. A doubly clamped flexible piezoelectric energy harvester (FPEH) with axial excitation is therefore proposed for higher power output in a low-frequency vibration environment. Combining the Euler–Bernoulli beam theory and the D’Alembert principle, the differential dynamic equation of the doubly clamped energy harvester is derived, in which the excitation mode of axial load with pre-deformation is considered. A numerical solution of voltage amplitude and average power is obtained using the Rayleigh–Ritz method. Output power of 22.5 μW at 27.1 Hz, with the optimal load resistance being 1 MΩ, is determined by the frequency sweeping analysis. In order to power electronic devices, the converted alternating electric energy should be rectified into direct current energy. By connecting to the MDA2500 standard rectified electric bridge, a rectified DC output voltage across the 1 MΩ load resistor is characterized to be 2.39 V. For further validation of the mechanical-electrical dynamical model of the doubly clamped flexible piezoelectric energy harvester, its output performances, including both its frequency response and resistance load matching performances, are experimentally characterized. From the experimental results, the maximum output power is 1.38 μW, with a load resistance of 5.7 MΩ at 27 Hz, and the rectified DC output voltage reaches 1.84 V, which shows coincidence with simulation results and is proved to be sufficient for powering LED electronics.

## 1. Introduction

In recent years, flexible electronics have gained rapid attention for their prospective applications in organ implant surgery, soft robotics, and biomechanics. The pressing demand for seeking alternative solutions to replace bulky batteries to power in situ medical monitoring devices, soft robots and wearable electronics with unlimited battery life has led to tremendous research effort regarding flexible energy harvesters [1,2,3] due to its advantages of being lightweight and flexible, with a high energy density [4,5,6]. Flexible energy harvesting devices can be categorized into three types: photovoltaic energy harvester, thermoelectric energy harvester, and mechanical-electric energy harvester. With regard to mechanical-electric energy harvesters, piezoelectric, triboelectric, electromagnetic, and electrostatic energy converting mechanisms have been examined extensively among which piezoelectric flexible energy harvesters have been widely utilized by converting mechanical energy from a stretchable surface into electrical energy [7,8]. The power generation performance of the piezoelectric vibration energy harvester largely depends on its geometric structure [9]. Although the cantilever beam structure has been extensively examined in energy harvesting devices, it needs additional proof mass to lower its resonant frequency to adapt ambient vibration. Quattrocchi [10,11] proposed a cantilever-type piezoceramic energy harvester which is attached with proof mass at the free end. In their work, different resonance configurations, as well as electrical and dynamical behavior were characterized—the resonant frequency was around 17 Hz.

As the resonant frequency is related to both mass and stiffness, the alternative way to harness low frequency vibration energy is to utilize the doubly clamped flexible beam structure, since it presents characteristics of low stiffness. Therefore, this paper focuses on taking the first step towards this vision, by exploring a doubly clamped flexible energy harvester that is able to convert mechanical energy to electric power from bending or stretching modes.

Since the piezoelectric energy harvester’s (PEHs) performance is closely related to its geometric structure and mechanical response, from the literature review, various models on PEHs with doubly clamped configurations have been investigated. In 2012, Cottone F [12] proposed a bistable non-magnetic piezoelectric buckled beam for random vibration energy harvesting by exerting an increasing axial compression. Compared to the unbuckled state, the device exhibited superior power generation over a large interval of resistive load, with gains up to more than a factor of ten. In 2014, Zhu Liang et al. [13] used a lumped parameter method to derive a serially connected doubly clamped beam model. By adjusting the asymmetry distance between the proof and supporting mass, the device can generate a power of 0.8 mW from 144 Hz to 170 Hz. In 2015, Zhu Liang et al. [14] derived theoretical models for both a unimorph and bimorph piezoelectric doubly clamped beam energy harvester through the energy method. In 2016, Zhou Z [15] presented a nonlinear flexible bistable energy harvester that can realize snap-through by a clamped-clamped beam structure with a mid-magnet to broaden the frequency bandwidth. Ahmed Emad et al. [16] utilized stretching strain of a Polyvinylidene Fluoride (PVDF) doubly clamped piezoelectric beam structure to harvest energy from vibrations, which exhibits a highly nonlinear frequency response that widens the bandwidth. In their result, the device with a design of a 9.9 mm^3^ energy harvester can generate up to 4 μW from vibrations of 0.5 g at 70 Hz. Kashyap R [17] utilized both stretching and bending strains by planting doubly clamped beams over an elastic base beam to enhance the power generation. Cui Y [18] presented a work on the fabrication and characterization of clamped-clamped piezoelectric energy harvesting devices with MEMS processes. By serially connecting doubly clamped beams, the maximum output voltage with an optimal load resistance reached 730 nW.

In order to match with low frequency, low amplitude, and unpredictable vibration source, Xu R [19] developed a bistable buckled beam energy harvester at the MEMS scale, which demonstrated 50% bandwidth under 70 Hz at 0.5 g input through dynamic testing. Su Weijun [20] developed a non-linear theoretical model for a bi-directional vortex-induced piezoelectric energy harvester with magnetic interaction based on the Euler–Bernoulli beam theory. Qin Y [21] built a distributed-parameter dynamic model of a bridge-shaped piezoelectric energy harvester, based on the Euler–Bernoulli beam hypothesis and the Hamilton principle. By moving the proof mass, it can broaden the frequency band and adaptively adjust the natural frequency of the system to match external excitation. In 2020, Yenuganti S [22] used a doubly clamped beam as an elastic structure for ambient low power excitation vibrational energy harvesting, which introduced a cavity along its thickness direction. Both analytical and numerical analyses revealed that the clamped-clamped micro beam (CCMB) with cavity yield a higher output voltage as compared to the CCMB without cavity. Li M [23] presented a pre-compressed cruciform energy harvester to effectively adjust the overall equivalent mass and stiffness so that it can reduce resonant frequency, improve voltage peak, and widen usable bandwidth. In their work, the mechanical model of the novel structure is developed to characterize the effects of geometry and a pre-compressed force on output performance, based on both the Euler–Bernoulli beam theory and finite element analysis. Alcheikh N [24] investigates the dynamics of a clamped–clamped straight multiple-steps micro-beam with constant width based on the Euler–Bernoulli beam theory and the Galerkin discretization. The results highlight the capability of the novel device that can tune the natural frequency by varying the geometry, the axial force, and nonlinear electrostatic forces.

The doubly clamped piezoelectric energy harvesters aforementioned mainly focused on the lateral excitation, which is usually attached with proof masses to lower the working frequency. However, the alternative way to reduce the resonant frequency is to utilize the plastic substrate. In this paper, a doubly clamped beam energy harvester with flexible plastic substrate without proof mass is devised. For further improvement of the output performance, the doubly clamped structure is sinusoidally buckled by axial load. The analytical model is built based on the Euler–Bernoulli beam theory considering the axial excitation and solved by the Rayleigh–Ritz method. In order to further validate the efficiency, output performance of the doubly clamped flexible piezoelectric energy harvester, connected with a standard rectifier circuit, is experimentally characterized.

The rest of the paper is organized as follows. Section 2 develops an analytical mechanical-electrical coupling model for doubly clamped PEHs with axial excitation. Numerical simulations of the analytical model are implemented in Section 3. In Section 4, a series of experiments with prototype devices are performed to verify the models from the perspective of frequency response and resistance load matching. Conclusion remarks are drawn in Section 5.

## 2. Analytical Model of Doubly Clamped Flexible PEH

### Analytical Model Based on Euler–Bernoulli Beam Theory

**Figure 1:**
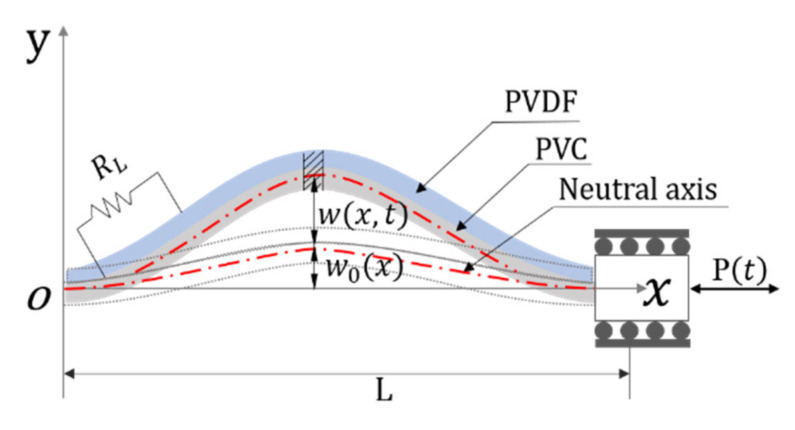
Schematic diagram of the doubly clamped piezoelectric energy harvester.

Schematic configuration of the doubly clamped PEH is shown in Figure 1, which is composed of a unimorph piezoelectric film (PVDF) attached on a flexible substrate with a pre-deformation. Polyvinyl Chloride (PVC) film, with elongation at break being about 100–400%, and flexural modulus ranging from 0.001 GPa to 1.8 GPa, is chosen as the flexible substrate. One end of the device is fixed, and the other end is clamped and sinusoidally excited by axial external force input *P*(*t*).

**Figure 2:**
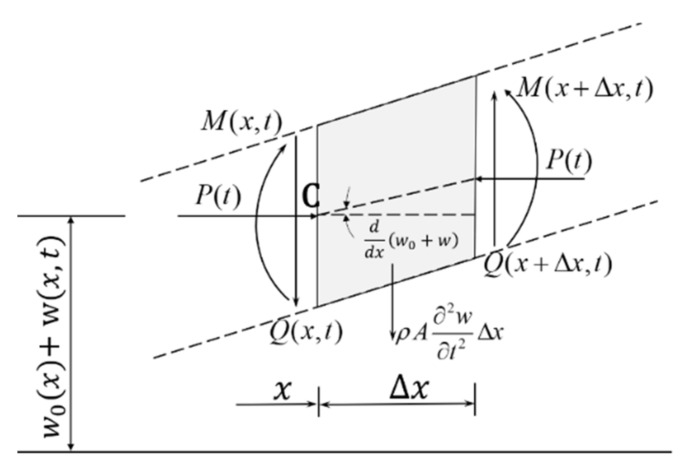
Deformation of a finite beam element in PEH.

In the beam model, the origin is set at the centroid of the left end. The *y*-axis is set upward along the thickness, and the *x*-axis is set to the right direction along the neutral plane. Assuming that the central principal inertia axis of every beam element section is in *xy* plane, the piezoelectric beam is pre-compressed with an initial deflection, which is represented by *w*_0_(*x*). According to D’Alembert’s principle, it is supposed that forces in the finite beam element acting at point *C*. As shown in Figure 2, the external load is applied horizontally so that the beam is buckled to induce transverse deformation.

Based on the classical Euler–Bernoulli beam theory, the shear deformation, and the inertia of the section on the neutral axis are ignored and the transverse deflection *w*(*x*, *t*) is considered in the force and moment dynamic equilibrium Equation (1), where *Q*(*x*, *t*) is the shear force, *ρ* is the density of composite beam, *A* is the cross-sectional area. *M*(*x*, *t*) represents the force moment, and *P*(*t*) is the axial dynamic load.

(1) $$\left\{\begin{array}{c} Q(x,t)-Q(x+\Delta x,t)-\rho A\Delta x\frac{\partial^{2}w}{\partial t^{2}}=0\\ M\left(x,t\right)-M\left(x+\Delta x,t\right)+\rho A\frac{\partial^{2}w}{\partial t^{2}}\Delta x\frac{\Delta x}{2}+Q\left(x+\Delta x,t\right)\times \Delta x-P\left(t\right)\frac{\partial \left(w+w_{0}\right)}{\partial x}\times \Delta x=0 \end{array}\right.$$

If higher-order terms of Δ*x* is ignored, the dynamic Equation (1) can be derived as Equation (2).

(2) $$\frac{\partial^{2}M\left(x,t\right)}{\partial x^{2}}+P\left(t\right)\frac{\partial^{2}\left(w+w_{0}\right)}{\partial x^{2}}+\rho A\frac{\partial^{2}w\left(x,t\right)}{\partial t^{2}}=0$$

If viscous damping forces, including both external damping force and internal resistance, in the piezoelectric patch are considered in Equation (2), the dynamic equation can be rewritten as formula (3).

(3) $$\frac{\partial^{2}M(x,t)}{\partial x^{2}}+P(t)\frac{\partial^{2}}{\partial x^{2}}\left[w\left(x,t\right)+w_{0}\left(x\right)\right]+C_{s}I\frac{\partial^{5}w(x,t)}{\partial x^{4}\partial t}+C_{a}\frac{\partial w(x,t)}{\partial x}+\rho A\frac{\partial^{2}w\left(x,t\right)}{\partial t^{2}}=0$$

In the above equation, *C*_a_ is the strain damping coefficient for external distributed damping force, and *C_s_* denotes equivalent strain rate damping coefficient. The distributed external damping force is proportional to differential transverse deflection *w*(*x*, *t*), which is expressed as Equation (4).

(4) $$f_{D}\left(x,t\right)=C_{a}\frac{\partial w\left(x,t\right)}{\partial t}$$

Since the damping stress changes linearly in the height direction in the cross-section, the damping moment can be deduced as Equation (5) from the damping stress.

(5) $$M_{D}=\iint_{A}\sigma_{D}ydA=C_{s}I\frac{\partial^{3}w}{\partial x^{2}\partial t}$$

where *I* is the equivalent area moment of inertia for the cross section of composite beam.

From the constitutive equations, the stresses *T* of elastic substrate and piezoelectric layer are expressed, respectively, as (6) and (7)

(6) $$T_{1}^{s}=Y_{s}s_{1}^{s}$$

(7) $$T_{1}^{p}=Y_{p}(s_{1}^{p}-d_{31}E_{3})$$

where $s_{1}^{s}$ and $s_{1}^{p}$ are the strain of elastic substrate and piezoelectric layer along the *x*-axis direction. Here, subscript/superscript *p* and *s* stand for PVDF and substructure layers, respectively. 1 and 3 directions are coincident with *x* and *y* directions, correspondingly. *Y* is Young’s modulus, and *E*_3_ denotes the electric field along the polarization direction. *d*_31_ is the piezoelectric coefficient.

The stress $s_{1}$ equals to the second derivative of transverse deflection *w*(*x*, *t*) with respect to position *x*, which is shown as Equation (8),

(8) $$s_{1}=-h_{pc}\frac{\partial^{2}w\left(x,t\right)}{\partial x^{2}}$$

Therefore, the internal moment of the cantilever *M* can be written as (9)

(9) $$M\left(x,t\right)=-\int_{h_{a}}^{h_{b}}T_{1}^{s}bydy-\int_{h_{b}}^{h_{c}}T_{1}^{p}bydy$$

**Figure 3:**
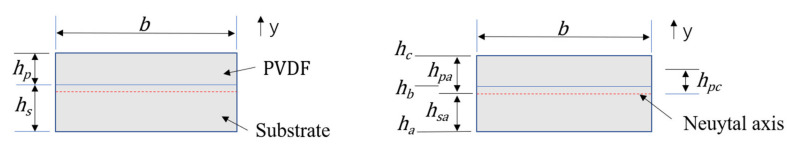
Geometric dimensions of the flexible beam section.

The thicknesses of the beam and piezoelectric layer are *h*_s_ and *h*_p_, respectively, and the width for both layers are the same as *b* (shown in Figure 3). *h*_c_ is the position of the top of PVDF layer from the neutral axis. *h*_b_ represents the position of the bottom of PVDF layer from the neutral axis. *h*_a_ denotes the position of the bottom of the substrate layer. *h*_pa_ are distances between the top of piezoelectric layer and neutral axis, while *h*_sa_ denotes the distance between the substrate bottom and neutral axis. *h*_pc_ is the distance between center line of piezoelectric layer and neutral axis.

According to the geometric relationship among *h*_s_, *h*_p_, *h*_a_, *h*_b_ and *h*_c_, the following expressions can be deduced:

(10) $$\left\{\begin{array}{l} h_{a}=-\frac{Y_{s}h_{s}^{2}+2h_{s}Y_{p}h_{p}+Y_{p}h_{p}^{2}}{2Y_{p}h_{p}+2Y_{s}h_{s}}\\ h_{b}=\frac{Y_{p}h_{p}^{2}+2Y_{s}h_{p}h_{s}+Y_{s}h_{s}^{2}}{2(Y_{p}h_{p}+Y_{s}h_{s})}-h_{p}\\ h_{c}=-\frac{Y_{p}h_{p}^{2}+2Y_{s}h_{p}h_{s}+Y_{s}h_{s}^{2}}{2(Y_{p}h_{p}+Y_{s}h_{s})} \end{array}\right.$$

By substituting Equations (6) and (7) into (9), the internal moment *M*(*x*,*t*) can be expressed as (11):

(11) $$M\left(x,t\right)=b\frac{Y_{s}\left(h_{b}^{3}-h_{a}^{3}\right)+Y_{p}\left(h_{c}^{3}-h_{b}^{3}\right)}{3}\frac{\partial^{2}w\left(x,t\right)}{\partial x^{2}}+\frac{Y_{p}d_{31}b}{h_{p}}\left(h_{c}^{2}-h_{b}^{2}\right)v\left(t\right)$$

where *v*(*t*) is denoted as the voltage across the PVDF layer that can be deduced from the electric field *E*_3_(*t*) = *v*(*t*)/*h*_p_. For simplification of Equation (11), the coefficient of the first term can be represented as (*YI*)*_com_*, which is expressed as (12),

(12) $${\left(YI\right)}_{com}=Y_{p}I_{p}+Y_{s}I_{s}=b\frac{Y_{s}\left(h_{b}^{3}-h_{a}^{3}\right)+Y_{p}\left(h_{c}^{3}-h_{b}^{3}\right)}{3}$$

The coefficient of second term can be represented by *ϑ*, which is expressed as formula (13).

(13) $$\vartheta =-\frac{Y_{p}d_{31}b}{h_{p}}\left(h_{c}^{2}-h_{b}^{2}\right)$$

Then Equation (11) can be rewritten as (14)

(14) $$M\left(x,t\right)=YI\cdot \frac{\partial^{2}w\left(x,t\right)}{\partial x^{2}}+\vartheta v\left(t\right)$$

By integrating Equation (14) in Equation (3), it yields formula (15)

(15) $$\begin{array}{l} YI\frac{\partial^{4}w\left(x,t\right)}{\partial x^{4}}+P(t)\frac{\partial^{2}w\left(x,t\right)}{\partial x^{2}}+C_{s}I\frac{\partial^{5}w(x,t)}{\partial x^{4}\partial t}+C_{a}\frac{\partial w(x,t)}{\partial x}\\ +\rho A\frac{\partial^{2}w\left(x,t\right)}{\partial t^{2}}+\vartheta v\left(t\right)=-P(t)\frac{\partial^{2}w_{0}\left(x,t\right)}{\partial x^{2}} \end{array}$$

Equation (15) describes the dynamic behavior the flexible doubly clamped energy harvester that considers both effects from axial force and electromechanical coupling mechanism. Furthermore, from the second term of piezoelectric constitutive equation, the electric displacement *D*_3_ is denoted as (16)

(16) $$D_{3}(x,t)=d_{31}T_{1}^{p}+\varepsilon_{33}^{T}E_{3}$$

where $\varepsilon_{33}^{T}$ is the constant-stress dielectric constant, which can be replaced by the permittivity at constant strain, that is $\varepsilon_{33}^{T}=\varepsilon_{33}^{s}+d_{31}^{2}Y_{p}$. Then Equation (16) can be rewritten as (17)

(17) $$D_{3}(x,t)=d_{31}Y_{p}s_{1}-\varepsilon_{33}^{s}\frac{v\left(t\right)}{h_{p}}$$

If the doubly clamped beam is simplified to be the quarter symmetric model, electrical charge *q*(*t*) can be obtained by integrating the electrical displacement over the electrode area with:

(18) $$q\left(t\right)=\int_{A}D\cdot ndA=4\int_{x=0}^{\frac{L}{4}}\left(-bd_{31}Y_{p}y\frac{\partial^{2}w\left(x,t\right)}{\partial x^{2}}-\varepsilon_{33}^{s}b\frac{v\left(t\right)}{h_{p}}\right)dx$$

where **D** is the vector of electric displacements and *n* is the unit normal. The current can be obtained by the first differential of electrical charge with respect to time *t*,

(19) $$i\left(t\right)=\frac{dq\left(t\right)}{dt}=4\left(-\int_{x=0}^{\frac{L}{4}}bd_{31}Y_{p}y\frac{\partial^{2}w\left(x,t\right)}{\partial x^{2}\partial t}dx-\frac{\varepsilon_{33}^{s}bL}{4h_{p}}\frac{dv\left(t\right)}{dt}\right)$$

The output voltage across the resistive load is given by: *v*(*t*) = *R*_L_ × *i*(*t*)

(20) $$v\left(t\right)=R_{L}i\left(t\right)=4\left(-\int_{x=0}^{\frac{L}{4}}R_{L}bd_{31}Y_{p}y\frac{\partial^{2}w\left(x,t\right)}{\partial x^{2}\partial t}dx-\frac{\varepsilon_{33}^{s}bLR_{L}}{4h_{p}}\frac{dv\left(t\right)}{dt}\right)$$

The electrical equation can be represented by formula (21)

(21) $$\frac{v\left(t\right)}{R_{L}}+\frac{\varepsilon_{33}^{s}bL}{h_{p}}\frac{dv\left(t\right)}{dt}=4bd_{31}Y_{p}\int_{x=0}^{\frac{L}{4}}\frac{\partial^{2}w\left(x,t\right)}{\partial x^{2}\partial t}dx$$

Equations (15) and (21) are the distributed parameter electromechanical equations for PEHs clamped at both ends under axial excitation. Then, according to Rayleigh–Ritz method, the transverse deflection is rewritten as (22),

(22) $$w\left(x,t\right)=\sum_{r=1}^{\infty }\phi_{r}(x)\varphi_{r}(t)$$

where *ϕ*_r_(*x*) and *φ*_r_(*t*) are the mass normalized eigenfunction and vibration response of the *r*-th order mode of the doubly clamped beam model, respectively. Since the doubly clamped beam energy harvester is pre-deflected for sinusoidal excitation, the function of initial pre-deformation *w*_0_ (*x*) is obtained by curve fitting, shown as (23),

(23) $$w_{0}(x)=2.6\times 10^{4}x^{4}-2341\times x^{3}+55.97\times x^{2}-0.1481\times x$$

By considering boundary conditions for doubly clamped beams in Equation (15),

(24) $${\left.w\right|}_{x=0}={\left.w\right|}_{x=L}=0,{\left.\frac{dw}{dx}\right|}_{x=0}={\left.\frac{dw}{dx}\right|}_{x=L}=0$$

The normalized eigenfunction *ϕ*_r_(*x*) can be given by (25),

(25) $$\phi_{r}\left(x\right)=cosh\frac{\lambda_{r}}{L}x-cos\frac{\lambda_{r}}{L}x-\sigma_{r}\left(sinh\frac{\lambda_{r}}{L}x-sin\frac{\lambda_{r}}{L}x\right)$$

where *λ*_r_ is the dimensionless frequency number obtained from the characteristic equation that given by (26)

(26) $$cos\lambda_{r}cosh\lambda_{r}=1$$

*σ_r_* is expressed as

(27) $$\sigma_{r}=\frac{cosh\lambda_{r}-cos\lambda_{r}}{sinh\lambda_{r}-sin\lambda_{r}}$$

The mass normalized form of the eigenfunctions given by Equation (25) satisfies the following orthogonality conditions:

(28) $$\int_{0}^{L}\rho A\left(x\right)\phi_{s}\left(x\right)\phi_{r}\left(x\right)dx=\delta_{rs},\int_{0}^{L}\phi_{s}\left(x\right)YI\phi_{r}{}^{\left(4\right)}\left(x\right)dx=w_{r}^{2}\delta_{rs}$$

where δ*_rs_* is the Kronecker delta, defined to be equal to unity for *s* = *r* and equal to zero for *s* ≠ *r*. *w*_r_ is the undamped natural frequency of the *r*th mode given by

(29) $$\omega_{r}={\left(\lambda_{r}\right)}^{2}\sqrt{\frac{YI}{mL^{4}}}$$

Using Equation (22) in the partial differential equation of motion along with the orthogonality conditions given by Equation (28), the electromechanically coupled ordinary differential Equation (15) for the modal response of the beam can be obtained as (30)

(30) $$\frac{d^{2}\varphi_{r}\left(t\right)}{dt^{2}}+2\xi_{r}\omega_{r}\frac{d\varphi_{r}\left(t\right)}{dt}+\omega_{r}^{2}\varphi_{r}(t)+k_{r1}\varphi_{r}\left(t\right)P\left(t\right)+\vartheta \;v\left(t\right)=k_{r2}P\left(t\right)$$

where

(31) $$\xi_{r}=\frac{\omega_{r}C_{s}I}{2YI}+\frac{C_{a}}{2\rho A\omega_{r}}$$

(32) $$k_{r1}=\int_{0}^{L}\phi_{s}\left(x\right)\overset{..}{\phi_{r}}\left(x\right)dx$$

(33) $$k_{r2}=\int_{0}^{L}\frac{d^{2}w_{0}\left(x\right)}{dx^{2}}\phi_{s}\left(x\right)dx$$

In the same way, by substituting Equation (22) into (21), the equation expression of the output voltage across the resistive load can be written as (34)

(34) $$\frac{dV\left(t\right)}{dt}+\frac{1}{\tau_{c}}v\left(t\right)=\sum_{r=1}^{\infty }\eta_{r}\frac{d\varphi_{r}(t)}{dt}$$

where

(35) $$\eta_{r}=-4\frac{d_{31}Y_{p}h_{pc}h_{p}}{\varepsilon_{33}^{s}L}\int_{x=0}^{\frac{L}{4}}\frac{d^{2}\phi_{r}(x)}{dx^{2}}dx$$

(36) $$\tau_{c}=\frac{R_{L}\varepsilon_{33}^{s}bL}{h_{p}}$$

## 3. Numerical Simulation

According to Equations (30) and (34) obtained in Section 2, the dynamic characteristics of the doubly clamped piezoelectric energy harvester is numerically examined. Table 1 shows geometric dimensions, material properties and electromechanical coupling parameters of the energy harvester. Both the top and bottom surfaces of the PVDF piezoelectric layer are plated with electrodes. The harvester is excited by the axial harmonic force *P*(*t*) = *P*·*sin*(*ωt*) in the longitudinal direction at the right end.

### 3.1. Frequency Response

**Figure 4:**
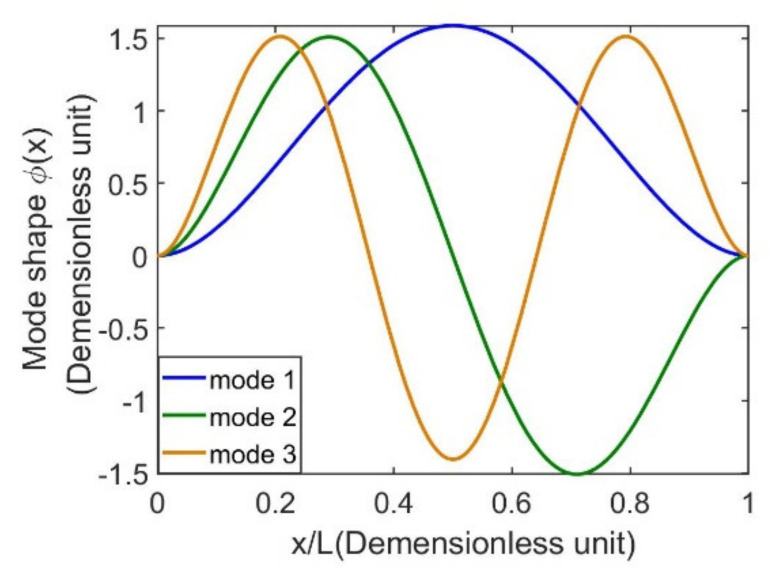
Normalized mode shapes of first three modes of doubly clamped beams.

In the simulation, the first mode damping ratio $\xi_{1}$ shown in (31) is defined to be 0.010. By substituting values of geometric dimensions, material properties, and electromechanical parameters shown in Table 1 into Equation (29), the undamped natural frequency at the first mode is calculated to be 27 Hz, 74 Hz and 146 Hz. Figure 4 shows the first, second and third mode shapes of the doubly clamped energy harvester, respectively.

**Figure 5:**
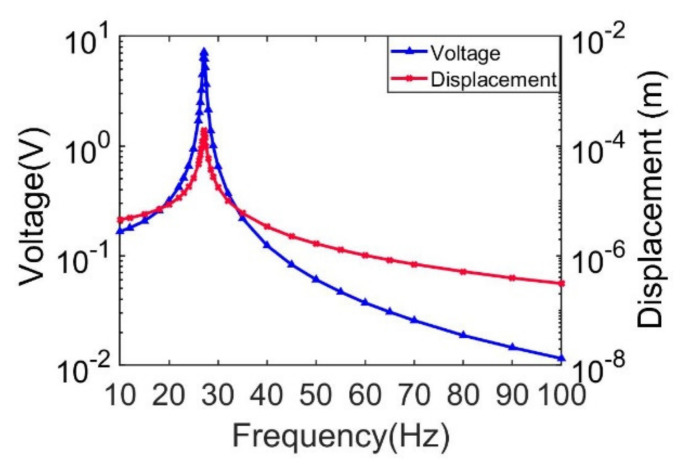
Simulation results of transverse deflection versus frequency at L/2.

**Figure 6:**
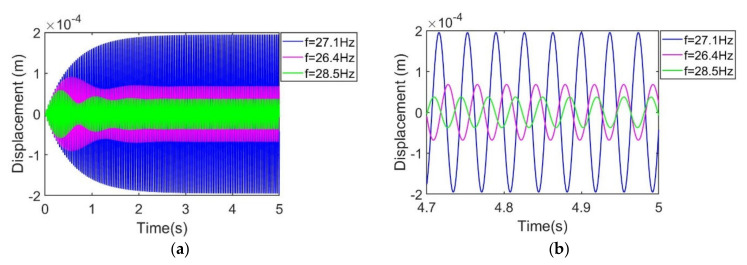
Transient simulation results of relative transverse deflection in (**a**) 5 s time scale and (**b**) 0.3 s time scale at steady status.

Among these three modes, the first-order mode is usually utilized. In order to characterize the dynamic response around the first mode, the doubly clamped beam is excited in the range of 10~100 Hz. Frequency response curves of transverse deflection and output voltage are obtained in Figure 5, combining Equations (30) and (34). It can be seen that both the output voltage and the transverse displacement present a Gaussian distribution, and the peak position corresponds to 27.1 Hz, where the peak output voltage is 7.2 V and the peak transverse deflection is 0.2 mm. Figure 6a shows the transient response of transverse deflection within 0~5 s under the excitation frequency of 26.4 Hz, 27.1 Hz and 28.5 Hz, respectively. It can be seen from the figure that after about 2 s, the transient transverse deflection presents a steady state in the form of a sinusoid. The transient response curves in the steady state are shown in Figure 6b, where transient transverse deflection amplitude is 0.2 mm with excitation frequency being 27.1 Hz. Transient transverse deflection amplitudes are 0.06 mm and 0.037 mm, corresponding to frequencies of 26.4 Hz and 28.5 Hz.

**Figure 7:**
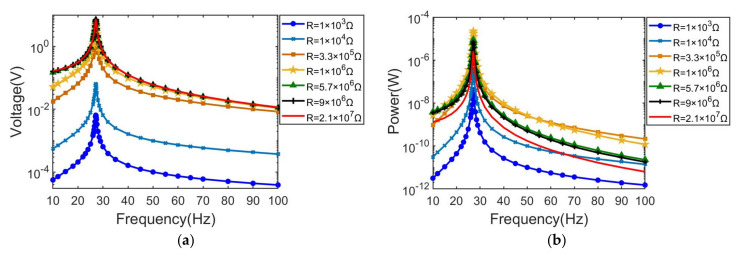
Simulation results of the effects of excitation frequency. (**a**) output voltage response with different load resistance; (**b**) average power response with different load resistance.

In order to further characterize the output performance, the optimal external load resistance can be determined through the frequency response functions (FRFs). By sweeping the frequency from 10 Hz to 100 Hz, results of the output voltage and power across seven load resistors ranging from 103 Ω to 107 Ω are plotted as shown in Figure 7a,b, respectively.

As can be seen in the Figure 7a, changing trends of output voltages across seven load resistors all present the Gaussian shape. Peak output voltages across external load resistors are all induced at 27.1 Hz. The peak output voltage in the gaussian shape curve increases monotonically with external load resistance, where the maximum peak output voltage is 7.2 V with the load resistance of 21 MΩ, and the minimum peak output voltage is 6.4 mV with the load resistance of 1 kΩ.

For plot results of the power output shown in Figure 7b, all changing trends also present the gaussian shape, while the peak value of the output power does not show a monotonic increase relationship with increasing load resistance. Among them, the maximum output power is 22.5 µW across the optimum load resistance of 1 MΩ at frequency ω = 27.1 Hz, and the minimum output power is 0.42 µW across the load resistance of 1 kΩ at frequency ω = 27.1 Hz.

**Figure 8:**
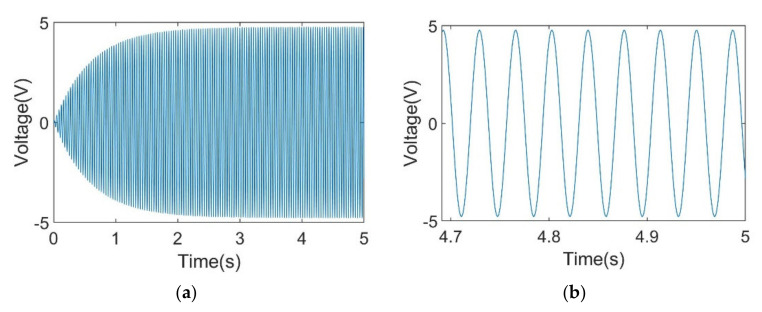
Simulation result of output voltage response in (**a**) a 5 s time scale and (**b**) a 0.3 s time scale at a steady status.

Figure 8 depicts simulation results of output voltage response with optimum load resistance at 27.1 Hz excitation with 5 s and 0.3 s time scales, respectively. From the results, it can be observed that amplitude of the output voltage at the optimum load resistance increases at the beginning. After about 4 s, the amplitude is steady at about 5 V, as shown in Figure 8b.

**Figure 9:**
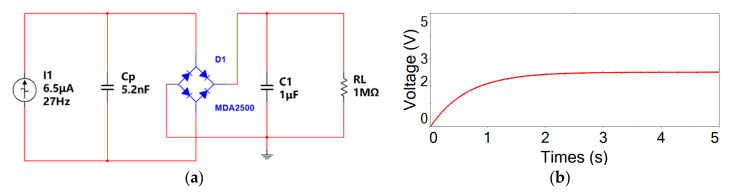
Simulation results of the doubly clamped flexible energy harvester with a standard electric circuit: (**a**) standard bridge electric circuit for the piezoelectric energy harvester; (**b**) output voltage across the load resistor.

Figure 8 shows the simulation result of the alternative output voltage response in the open electric circuit. However, the converted electric energy should be rectified into direct current for powering electronics. Figure 9 shows a schematic electric diagram of the standard rectified electric circuit and its corresponding simulation result of output voltage response. In the rectified electric circuit, the doubly clamped electric energy harvester is equivalent to an alternative current source with an amplitude of 6.5 µA and a frequency of 27 Hz. The internal capacitance of the scavenger is 5.2 nF. The MDA2500 bridge component is chosen as the standard rectified circuit bridge. A pair of electric bridge pins are connected with an equivalent power source, and the other pins are connected with a storage capacitor of 1 µF and a load resistor of 1 MΩ. From Figure 9b, it is shown that the rectified output voltage across the load resistor reaches up to 2.39 V in about 2 s.

### 3.2. Resistance Load Matching

**Figure 10:**
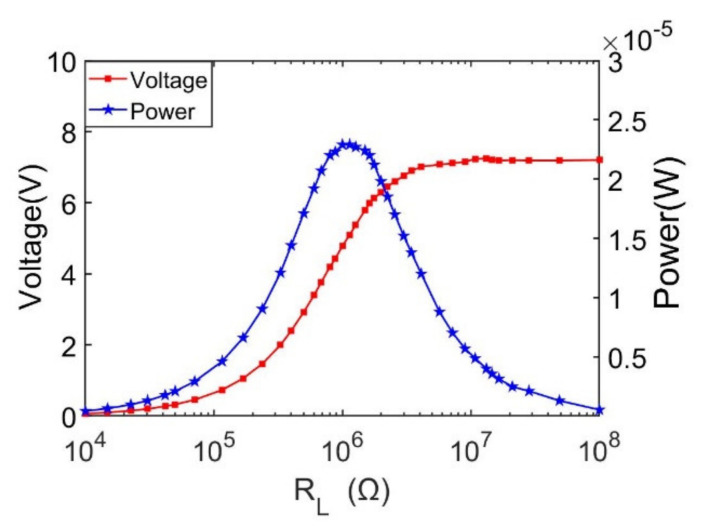
Simulation results of output voltage and power versus load resistance at first mode resonant frequency.

In order to achieve maximum output power, resistance load matching for the doubly clamped energy harvester is studied. The output performances, including both output voltage and power versus external load resistance ranging from 1 kΩ to 100 MΩ at 27.1 Hz, are characterized as shown in Figure 10. It can be found that output voltage reaches up to 7.19 V with a 16.5 MΩ external load resistance. As the external load resistance is continually increased, the voltage output stays almost at a steady value. It is proved by the output power trendline, as shown in Figure 10, that the peak output is 22.5 μW with an optimum load resistance of 1 MΩ.

### 3.3. Effects of Piezoelectric Layer Thickness on the Output Performanc of Energy Harvester

From Equation (29), it is observed that the resonant frequency is related to the equivalent stiffness (*YI*)_com_ of the composite beam. Formula (12) further reveals that the equivalent stiffness is determined by Young’s modulus, mass density and geometric dimensions of the flexible substrate and piezoelectric layer. When the material property is determined, geometric dimensions, especially the thickness of the piezoelectric layer, can play a significant role in the output performance of the energy harvester. In this section, the effect of the piezoelectric layer thickness on the output performance of the energy harvester is systematically examined. In the simulation, parameters, including excitation amplitude, Young’s modulus, mass density, substrate width, substrate thickness, substrate length, piezoelectric layer length and width, are kept constant. Then the relationship between natural frequency and thickness of the piezoelectric layer can be drawn as formula (37),

(37) $$\omega_{r}={\left(\lambda_{r}\right)}^{2}\sqrt{\frac{b}{3mL^{4}}\left(Y_{s}\left(h_{b}^{3}-h_{a}^{3}\right)+Y_{p}\left(h_{c}^{3}-h_{b}^{3}\right)\right)}$$

**Figure 11:**
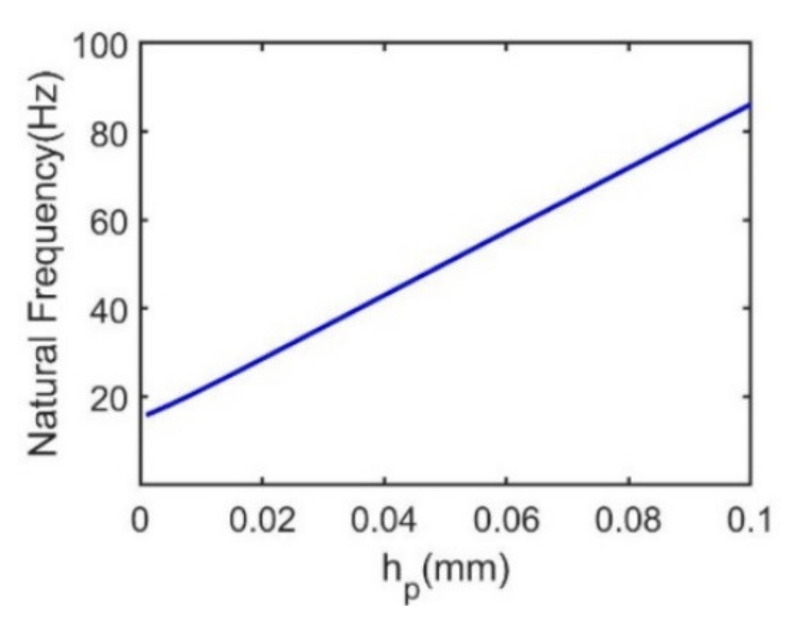
Relationship between natural frequency and piezoelectric layer thickness.

In the formula, *h*_a,_ *h*_b_ and *h*_c,_ can all be expressed by the piezoelectric layer thickness *h*_p,_ as it is shown in formula (10). Therefore, by changing this thickness from 0.01 mm to 0.1 mm, the relationship between natural frequency and the piezoelectric layer thickness is characterized as shown in Figure 11. It is observed that the natural frequency increases linearly with the piezoelectric layer thickness. When the piezoelectric layer thickness is varying from 0.001 mm to 0.1 mm, the natural frequency is increased from 15.8 Hz to 86.1 Hz.

**Figure 12:**
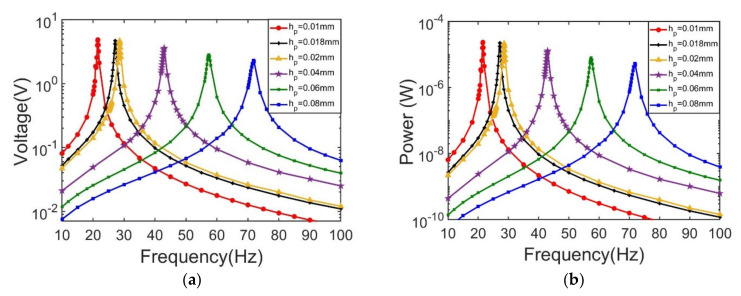
Frequency responses of output voltage and output power at first vibration mode. (**a**) output voltage responses with different piezoelectric layer thicknesses; (**b**) output power responses with different piezoelectric layer thicknesses.

Frequency responses of output voltage and output power with different piezoelectric layer thicknesses are shown in Figure 12a,b. It is observed that as the piezoelectric layer thickness increases, the resonant frequency is monotonically increased from 21.5 Hz to 71.7 Hz, while the peak output voltage is decreased from 4.87 V to 2.32 V at 1 MΩ, and output power drops down from 21 μW to 5.3 μW, correspondingly.

**Figure 13:**
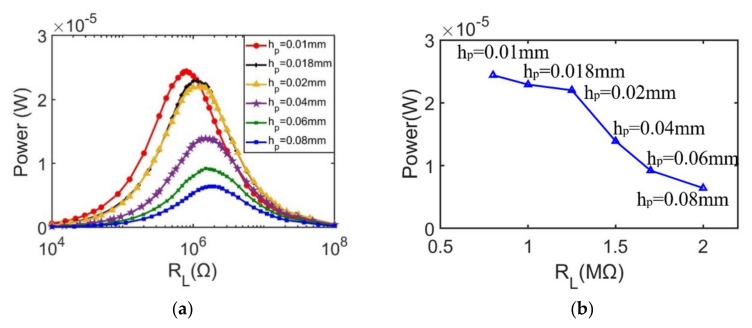
Resistance load matching results at the first vibration mode with different piezoelectric layer thickness. (**a**) output power responses with different piezoelectric layer thickness; (**b**) The maximum output power versus optimal load resistance corresponding to different piezoelectric layer thicknesses.

Since optimal load resistance is determined by natural frequency and capacitance of piezoelectric layer through $R_{load}=1/\left(\omega_{r}C\right)$, and capacitance is determined by $C=\varepsilon Lb/h_{p}$, it is found that varying piezoelectric layer thickness not only alters the resonant frequency of the energy harvester, but also changes the optimal load resistance. Thus, the resistance load matching mechanism is characterized as shown as Figure 13. From the results, it can be observed that when the thickness is reduced from 0.08 mm to 0.01 mm, the maximum output power increases from 6.3 μW to 24 μW, while the optimal load resistance decreases from 2 MΩ to 0.8 MΩ.

## 4. Experimental Demonstration

### 4.1. Test. Setup for the Doubly Clamped Energy Harvester

**Figure 14:**
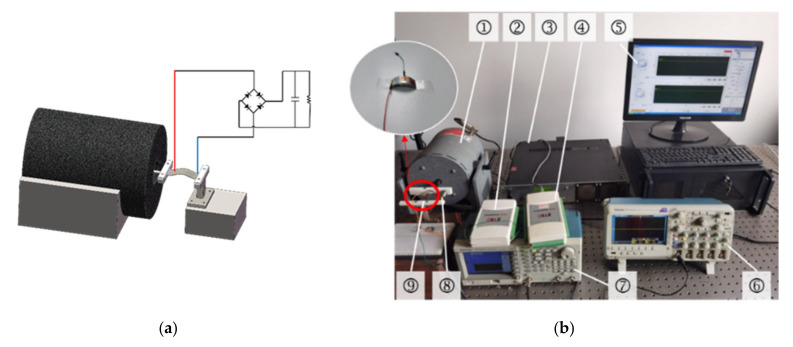
(**a**) a 3D model of the doubly clamped PEH; (**b**) measurement system of the doubly clamped energy harvester, in which ① is the vibrating shaker, ② is the signal conditioner, ③ is power amplifier, ④ is the data acquisition card, ⑤ is the computer, ⑥ is the oscilloscope, and ⑦ is the signal generator, ⑧ is doubly clamped energy harvester; ⑨ is the accelerometer.

In order to experimentally characterize the output performance of the proposed doubly clamped piezoelectric energy harvester, a prototype is fabricated, as shown in Figure 14, which consists of a piezoelectric film (PVDF) and a flexible PVC substrate with a certain pre-deformation. As shown in Figure 14a, one end of the energy harvester is fully fixed, and the other end is clamped by the clamper so that it can be excited with an axial force, through which the piezoelectric beam can be buckled into a curved shape.

As shown in Figure 14b, the doubly clamped piezoelectric energy harvester test system consists of a signal generator (manufacturer: Tektronix), power amplifier (manufacturer: MB Dynamics), vibration exciter (manufacturer: MB Dynamics), oscilloscope (manufacturer: Tektronix), accelerometer (manufacturer: Sinocera Piezotronics, inc), signal conditioner (manufacturer: ZTIC), data acquisition card (manufacturer: ZTIC), and PC computer. In the experiment, a signal generator AFG 3021C is used to generate sinusoidal excitations with different vibration amplitudes and frequencies. The excitation produced by the signal generator is then inputted to the amplifier, MB500VI, to drive the vibration shaker Model 50 A. The movable end of the doubly clamped energy harvester prototype is connected to the vibration shaker, which can be seen in Figure 14a. When the doubly clamped energy harvester is excited axially, the flexible piezoelectric beam will be buckled dynamically. The vibration signal is collected by an accelerometer and a data acquisition card. All the data will then be displayed on either the oscilloscope or the computer software.

### 4.2. Experimental Results

In order to verify the simulation results, output performance of the doubly clamped piezoelectric energy harvester, including frequency response and resistance load matching, is characterized.

#### 4.2.1. Frequency Response

**Figure 15:**
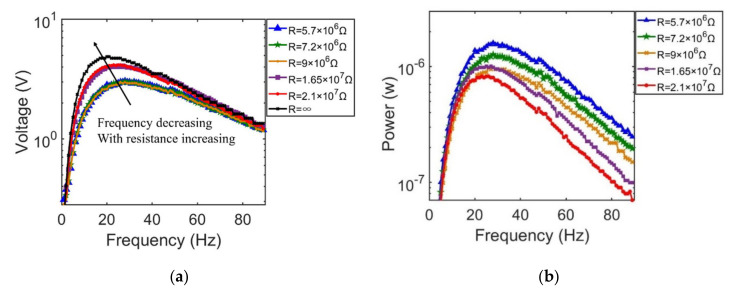
The results of the effects of excitation frequency for the first vibration mode. (**a**) output voltage response for different load resistance; (**b**) average power response for different load resistance.

In the experiment, output voltage and power responses are systematically investigated when the excited frequency is swept from _1_ Hz to 90 Hz, corresponding to different external load resistances, which are 5.7 MΩ, 7.2 MΩ, 9 MΩ, 16.5 MΩ and 21 MΩ. The corresponding output voltage frequency response curves are illustrated in Figure 15a. Compared to Figure 7a, an obvious larger bandwidth appears in experimentally measured response curves. However, in terms of the voltage change trend, the experimental voltage response curves are in good agreement with the simulation results, where both resonant frequencies are 27 Hz. Meanwhile, as the load resistance increases from 10^5^ Ω to 10^7^ Ω, the bandwidths in the experiment are significantly broadened in comparison to the simulation results. If load resistors are connected to the energy harvester, the main resonant frequency may gradually decrease from 27 Hz to 21 Hz with resistances increasing from 5.7 MΩ to an open circuit, which indicates that an axial load or pre-deformation for a doubly clamped energy harvester is not only suited for harnessing low frequency mechanical energy, but can also be adapted for wide band vibration energy sources.

Figure 15b shows the average power response for different load resistances. It is observed that the average power changing trends are the same as the simulation results. The peak output power is 1.55 μW with a load resistance of 5.7 MΩ at 27 Hz.

#### 4.2.2. Resistance Load Matching

**Figure 16:**
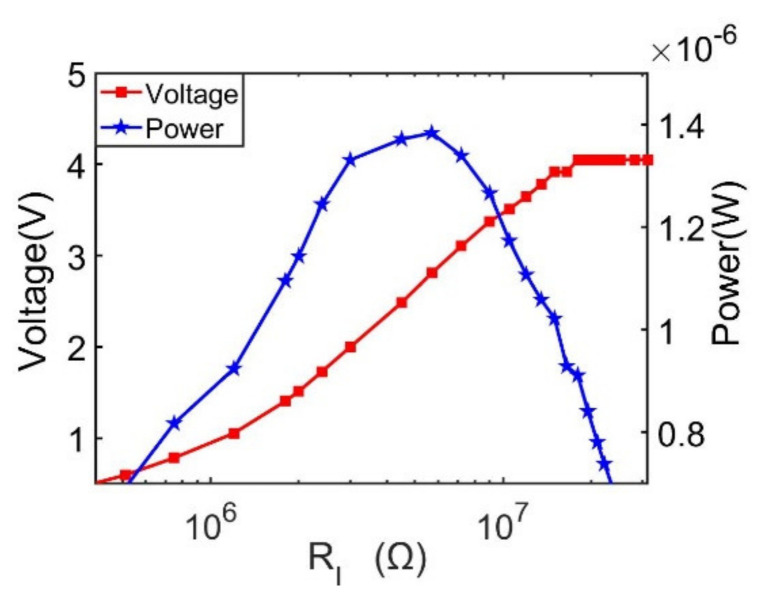
Resistance load matching results at the first vibration mode.

Impedance matching of the proposed doubly clamped piezoelectric energy harvester is carried out when the excitation frequency is 27 Hz. The output voltage and power curves versus the load resistance are shown in Figure 16. It can be seen that the output voltage increases with load resistance, increasing from 510 kΩ to 21 MΩ, where the maximum output voltage is 4.05 V at 21 MΩ. The output power trend-line presents a gaussian-like shape, which shows varying trends similar to those depicted in Figure 10. The output power first increases from 510 kΩ to 5.7 MΩ, and then decreases from 5.7 MΩ to 21 MΩ. From the results, we obtain that the maximum output power is 1.38 μW, corresponding to a 5.7 MΩ load resistance. Although the output power presents the same changing trend as shown in Figure 10, the optimal load resistance of 5.7 MΩ in the experiment is different, with a simulation result of 1 MΩ. This disagreement may have been caused by the mismatch of piezoelectric material property and the thickness measurement between the ideal simulation condition and actual experiment situation.

**Figure 17:**
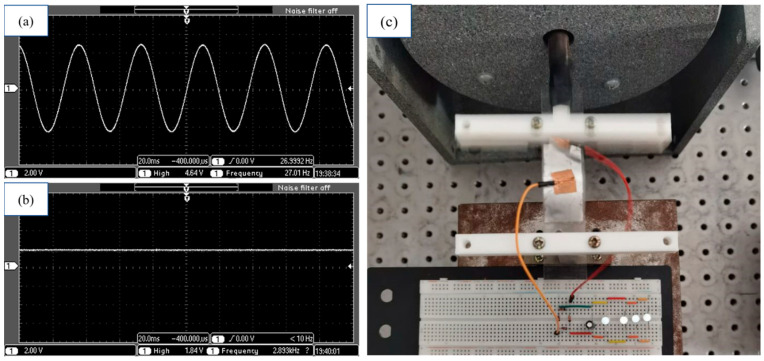
(**a**,**b**) are waveforms before and after rectification captured by the oscilloscope, (**c**) applications of the energy harvester to power up LEDs.

Figure 17a depicts the open circuit output voltage signal that is captured by the oscilloscope, where the amplitude is about 4.64 V at 27 Hz. Figure 17b shows the rectified output voltage signal, where DC output voltage is 1.84 V. In order to prove its prospective applications in powering electronics, the proposed doubly clamped energy harvester is used for powering light emission diodes (LEDs), Figure 17c shows the possibility of the flexible doubly clamped energy harvester to power four LEDs.

### 4.3. Comparison of Recent Doubly Clamped Energy Harvesters

Table 2 shows the comparison of output performances among recently developed doubly clamped energy harvesters, where resonant frequency, load resistance, output power, output voltage, overall size and power density are shown in the last five years.

Among references [18,19,21,23], although these works all propose a doubly clamped structure for vibration energy harvesters, proof masses are all attached to lower resonant frequencies catering for ambient vibration. Through this method, the corresponding resonant frequencies are lowered to 580 Hz, 70 Hz, 37 Hz and 120 Hz, as shown in Table 2. However, if the proof mass is not attached, the resonant frequency of the doubly clamped energy harvester can reach up to several kilohertz, as reference [24] cited. When compared with their results, the energy harvester proposed in this paper, with a flexible substrate under the axial excitation mode, presents the lowest resonance frequency (27 Hz). In addition, the matched external load resistance in this paper is 5.7 MΩ, which is much higher than in other works. The output voltage and power are 4.05 V and 1.38 μW with the volume being 36.9 mm^3^. Compared with other works, the output power density (0.037 μW/mm^3^) in this work is the highest, which is two orders of magnitude higher than [18] and one order of magnitude higher than [21]. From these viewpoints, it is indicated that the proposed doubly clamped flexible piezoelectric energy harvester is effective to scavenge low frequency vibration energy in ambient environments, which shows great potential for powering LEDs, sensor nodes, i-watches, and other wearable electronics.

## 5. Conclusions

In this paper, the analytical modeling of a doubly clamped flexible piezoelectric energy harvester under axial excitation force was put forward for mechanical energy harvesting. Combining both the Euler–Bernoulli beam theory and the D’Alembert principle, the differential dynamic equation of the doubly clamped pre-deformed beam is derived and numerical solutions to voltage and average power responses based on the Rayleigh–Ritz method are obtained. Through simulation analysis, the influence of excitation frequency and load resistance on the output voltage of the doubly clamped piezoelectric energy harvesting device is studied. It can be concluded that the output performance of the doubly clamped flexible energy harvester will be significantly improved when the frequency is around 27 Hz and the load resistance of 5.7 MΩ is matched. Finally, a prototype of the doubly clamped piezoelectric energy harvesting device was fabricated, and both frequency response and resistance load matching performances were tested. From the experimental results, the maximum output power is 1.38 μW, with a load resistance of 5.7 MΩ at 27 Hz, which is comparable with the simulation results and presents prominent advantages than other works. It is then proved that the doubly clamped structure is effective for the flexible energy harvester to capture energy from bending or stretching motions at low resonance frequencies, which shows its potential in applications for powering LEDs, sensor nodes, i-watches, and other wearable electronics.

## Figures and Tables

**Table 1:** Geometric dimensions, material properties, and electromechanical parameters of the flexible doubly clamped energy harvester.

Material and Geometric Parameters	Value/Unit
Geometry of the PVC substructure, *L* × *b* × *h*_s_	45 mm × 20 mm × 0.023 mm
Geometry of the the PVDF, *L* × *b* × *h*_p_	45 mm × 20 mm × 0.018 mm
Young’s modulus of the substructure, *Y*_s_	2.9 GPa
Young’s modulus of the PVDF, *Y*_p_	3.9 GPa
Mass density of the substructure, *ρ*_s_	1380 kg/m^3^
Mass density of the PVDF, *ρ*_p_	1780 kg/m^3^
Piezoelectric constant, *d*_31_	23 pm/V
Relative permittivity, *ε*/*ε*_0_	12

**Table 2:** Comparison of recent doubly clamped energy harvesters’ main characteristics.

Ref	Resonant Frequency (Hz)	Load Resistance (kΩ)	Mass	Output Voltage/Power	Overall Size (mm^3^)	Power Density (μW/mm^3^)	Time
[18]	580	60	✓	0.094 V/0.73 μW	2115.75	3.45 × 10^−4^	2018
[19]	70	1 × 10^3^	✓	−/0.08 μW	-	-	2019
[21]	37	300	✓	0.028 V/1.9 μW	708.5	2.68 × 10^−3^	2019
[23]	120	∞	✓	30 V/−	320.5	-	2020
[24]	20 k~140 k	∞		4.4 V~0.5 V/−	5 × 10^−6^	-	2020
This work	27	5.7 × 10^3^		4.05 V/1.38 μW	36.9	3.73 × 10^−2^	

## Data Availability

Not applicable.
